# Bioinformatic Integration of Molecular Networks and Major Pathways Involved in Mice Cochlear and Vestibular Supporting Cells

**DOI:** 10.3389/fnmol.2018.00108

**Published:** 2018-04-05

**Authors:** Teresa Requena, Alvaro Gallego-Martinez, Jose A. Lopez-Escamez

**Affiliations:** ^1^Otology & Neurotology Group CTS495, Department of Genomic Medicine—Centro de Genómica e Investigación Oncológica—Pfizer/Universidad de Granada/Junta de Andalucía (GENYO), Granada, Spain; ^2^Department of Otolaryngology, Instituto de Investigación Biosanitaria, ibs.GRANADA, Hospital Virgen de las Nieves, Universidad de Granada, Granada, Spain; ^3^Luxembourg Centre for Systems Biomedicine (LCSB), University of Luxembourg, Esch-sur-Alzette, Luxembourg

**Keywords:** hair cells, epithelial-non sensory cells, non-epithelial cells, cochlea, gene expression

## Abstract

**Background**: Cochlear and vestibular epithelial non-hair cells (ENHCs) are the supporting elements of the cellular architecture in the organ of Corti and the vestibular neuroepithelium in the inner ear. Intercellular and cell-extracellular matrix interactions are essential to prevent an abnormal ion redistribution leading to hearing and vestibular loss. The aim of this study is to define the main pathways and molecular networks in the mouse ENHCs.

**Methods**: We retrieved microarray and RNA-seq datasets from mouse epithelial sensory and non-sensory cells from gEAR portal (http://umgear.org/index.html) and obtained gene expression fold-change between ENHCs and non-epithelial cells (NECs) against HCs for each gene. Differentially expressed genes (DEG) with a log2 fold change between 1 and −1 were discarded. The remaining genes were selected to search for interactions using Ingenuity Pathway Analysis and STRING platform. Specific molecular networks for ENHCs in the cochlea and the vestibular organs were generated and significant pathways were identified.

**Results**: Between 1723 and 1559 DEG were found in the mouse cochlear and vestibular tissues, respectively. Six main pathways showed enrichment in the supporting cells in both tissues: (1) “Inhibition of Matrix Metalloproteases”; (2) “Calcium Transport I”; (3) “Calcium Signaling”; (4) “Leukocyte Extravasation Signaling”; (5) “Signaling by Rho Family GTPases”; and (6) “Axonal Guidance Si”. In the mouse cochlea, ENHCs showed a significant enrichment in 18 pathways highlighting “axonal guidance signaling (AGS)” (*p* = 4.37 × 10^−8^) and “RhoGDI Signaling” (*p* = 3.31 × 10^−8^). In the vestibular dataset, there were 20 enriched pathways in ENHCs, the most significant being “Leukocyte Extravasation Signaling” (*p* = 8.71 × 10^−6^), “Signaling by Rho Family GTPases” (*p* = 1.20 × 10^−5^) and “Calcium Signaling” (*p* = 1.20 × 10^−5^). Among the top ranked networks, the most biologically significant network contained the “auditory and vestibular system development and function” terms. We also found 108 genes showing tonotopic gene expression in the cochlear ENHCs.

**Conclusions**: We have predicted the main pathways and molecular networks for ENHCs in the organ of Corti and vestibular neuroepithelium. These pathways will facilitate the design of molecular maps to select novel candidate genes for hearing or vestibular loss to conduct functional studies.

## Introduction

**Figure 1:**
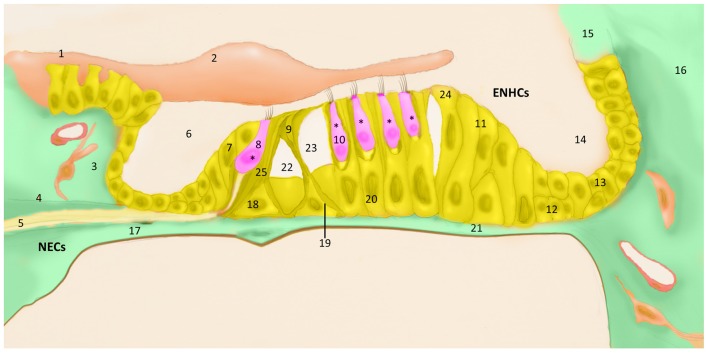
The organ of Corti cell populations; (1) grooves stripe, (2) tectorial membrane, (3) connective tissue, (4) bone spiral lamina, (5) nerve fibers, (6) cochlear ramp, (7) inner marginal cell, (8) inner hair cell (HC), (9) pillar cells of the tunnel of Corti, (10) outer HC, (11) Hensen cell, (12) Boettcher cell, (13) Claudius cell, (14) cochlear ramp, (15) stria vascularis, (16) cells of the spiral ligament, (17) mesothelium lining the tympanic ramp, (18) Inner Pillar cell (IPC), (19) outer pillar cell (OPC), (20) external phalangeal cell or Deiters cells, (21) basilar membrane (22), tunnel of Corti, (23) Nuel space, (24) tectal cells, (25) inner phalangeal cell (IPH). *Hair cells (HCs), pink; epithelial non-hair cells (ENCHs), yellow; non-epithelial cells (NECs), green.

The cellular architecture of the mammalian inner ear organs is complex, consisting of multiple types of polarized sensorineural epithelial cells, surrounded by supporting cells. The hearing organ, the cochlea, is located in the anterior labyrinth. It is a spiral, membranous tube with three divisions or scala (vestibular, media, tympani) around a central cone, the modiolus which contains the auditory nerve and the blood vessels. The organ of Corti is located in the scale media and there are more than 20 cell types arranged in close contact mediated by several types of intercellular junctions over the basilar membrane that maintain the homeostasis of the endolymph. Three or four rows of outer hair cells (HCs) lay over specialized supporting cells, the Deiters’ cells (outer phalangeal cells), and one row of inner HCs are surrounded by inner phalangeal cells (IPHs) and inner border cells in the organ of Corti (Pickles, 2015). The HCs have bundles of stereocilia in their apical surface, which are in contact with the tectorial membrane. Between them, Inner pillar cells (IPCs) and outer pillar cells (OPCs) forms the tunnel of Corti, and between OPC and the first row on outer HCs remains the space of Nuel. Towards the lateral wall, several non-sensory cells are found, including tectal cells, Hensen cells, Claudius cells and Boettcher cells, showing the structural complexity of the organ of Corti (Figure 1).

**Figure 2:**
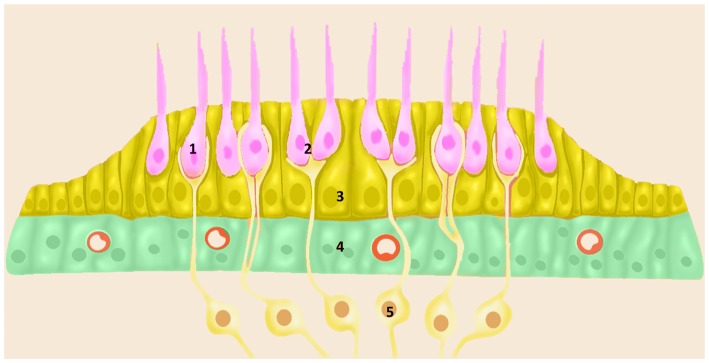
Schematic diagram of vestibular sensory epithelia (Crista; Saccule and Utricle) cell populations; (1) vestibular HC type I; (2) vestibular HC type II; (3) ENCH; (4) NECs; and (5) nerve fibers. Utricle epithelia, illustrating the cells designated as HCs (pink), ENHCs (yellow) and NECs (green) in each organ.

The posterior labyrinth hosts the vestibular system and it consists of five organs: the saccule and the utricle, sensors of lineal accelerations and the three semicircular canals: horizontal, anterior and posterior canals which sense angular accelerations. The vestibular sensory epithelium is composed of HCs and supporting cells. According to their shape and ultrastructure, there are two types of vestibular HCs (types I and II), both surrounded by supporting cells (Figure 2). Supporting cells extend from the basement membrane to the apical surface, and they present structural differences among the different vestibular organs (Lysakowski et al., 2011).

Gene expression studies in hair and supporting cells have been performed in chicken (Ku et al., 2014) and neonatal and adult mouse (Hertzano et al., 2011; Elkon et al., 2015), including single cell RNA expression studies showing tonotopic differences in supporting cells (Waldhaus et al., 2015).

Although there are significant morphological and functional differences between cochlear and vestibular supporting cells, there are some shared features that justify the comparison of their gene expression profiles: (a) the maintenance of the cellular architecture in the organ of Corti and the neurosensory epithelium in the vestibular sensory organs; (b) the existence of a system of intercellular junctions (Gulley and Reese, 1976; Taylor et al., 2015); and (c) a network of extracellular matrix proteins which interacts with the basal membrane (Santi and Johnson, 2013). These structures contribute to regulate endolymph homeostasis and prevent ion redistribution in the scala media resulting in loss of endocochlear potential.

The aim of this study is to define the main pathways in mice cochlear and vestibular non-sensory cells and to generate molecular networks maps to be used as backbone for the design of disease maps in hearing and vestibular disorder, including Meniere disease.

## Materials and Methods

### Raw and Pre-processed Data

We selected microarray and RNA-seq public datasets from postnatal day 0 and 1 mouse (P0 and P1) from auditory and vestibular epithelia. Both datasets are available in gEAR website^1^ (Table 1; datasets 1 and 2). These gene expression studies generated datasets for HCs, epithelial non-hair cells (ENHCs) and non-epithelial cells (NECs) of the organ of Corti and maculae vestibular epithelia separated by flow cytometry (Elkon et al., 2015).

**Table 1:** Description of gene expression datasets used in this study.

Data-set	Tissues	Stage	Specie	Cell/Tissue selection methods	RNA expression assays	References
1	Cochlea, vestibular system	P0–P1	Mouse	Atoh1/nGFP mice and Flow Cytometry	Microarray (MouseRef-6 v2.0 Expression BeadChips)	Elkon et al. (2015)
2	Cochlea, vestibular system	P0–P1	Mouse	Atoh1/nGFP mice and Flow Cytometry	Rnaseq (NuGEN Ovation RNA-seq V2 System & Ovation Ultralow Library System)	Elkon et al. (2015)
3	Cochlea	Adult (6 weeks)	Mouse	Manual dissection	Microarray (SurePrint G3 Mouse Exon Microarray)	Yoshimura et al. (2014)
4	Cochlea	P2	Mouse	Atoh1-nGFP, Sox2-GFP, Lgr5-EGFP-IRES-CreERT2, Fgfr3-CreERT2, Glast-CreERT2, Ai14-tdTomato mice and Flow Cytometry	Quantitative RT-PCR	Waldhaus et al. (2015)

These six cell subtypes were analyzed using Illumina MouseRef-6 v2.0 Expression BeadChips and basal expression levels were calculated using Illumina’s Bead-Studio package. Probes with *p*-value > 0.01 in at least two samples were discarded, and 23,051 probes corresponding to 17,275 genes remained (Elkon et al., 2015).

In addition, we collected processed gene expression data from P0–P2 and adult mouse cochlea, according to three turns of the cochlea (apical, middle and basal turns) to evaluate genes associated with tonotopy. The original datasets were generated using Agilent Mouse Exon Microarrays technology (Yoshimura et al., 2014) and quantitative RT-PCR (Waldhaus et al., 2015; Table 1; datasets 3 and 4).

Yoshimura et al. (2014) obtained RNA samples from each cochlear turn without cell distinction to generate a tonotopic gene expression profile. We selected genes showing at least 2-fold change between two cochlear turns. So, according 941 had >2-fold of 24,547 genes included in the microarray, but only 783 were annotated. From these 783 annotated genes; 747 were differentially expressed between apex and base; 51 were differentially expressed between apex and middle, and 458 genes were differentially expressed between middle and base. Furthermore, qPCR gene expression studies targeting 100 genes were performed in mice by Waldhaus et al. (2015) in 8 cell subtypes, previously sorted by cell type markers (Greater epithelial ridge (GER), Inner HC, Inner Pillar HC, IPH, OPC, Outer HCs, Dieters’ Cell row 1/2 and Dieters’ Cell row 3). We used this dataset to validate cochlear tonotopic gradient.

All procedures involving animals in the original studies were performed in accordance with institutional regulations and obtained the institutional animal care approval (Yoshimura et al., 2014; Elkon et al., 2015; Waldhaus et al., 2015).

### Cell Separation by Flow Cytometry

Atoh1/nGFP mice were used to obtain the different populations of cells by Elkon et al. (2015). So all HCs and neuron GFP(+) in the auditory and vestibular systems were selected. In addition, CD326 was used as epithelial cell marker in the inner ear. So, GFP(+) and CD326(+) cells were sorted as HCs; CD326(+) and GFP(−) were classified ENHCs, and cells negative both for GFP and CD326 were NECs. GFP(+)/CD326(−) neuronal cell were discarded (Elkon et al., 2015).

### Gene Expression Analysis

Gene expression fold-change between each cell type was calculated for each gene using Desq2 algorithm (Anders and Huber, 2010). Differentially expressed genes (DEG) with a log2 fold change between 1 and −1 were not considered significant and discarded for this study to reduce methodological artifacts.

### Cell Type Data Analysis

Data were normalized using quantile normalization. For the microarray dataset, a gene was considered as a “marker gene” of a given cell type and tissue, if it was expressed at a value >250 in the cell type of interest, and it had a value of <120 in all other samples according to Elkon et al. (2015). For RNA-seq data, a “marker gene” should have >50 reads in the cell type of interest and a value of <20 in all other samples. Data were normalized divided by the threshold of 250.

### Generation of Gene Pathways and Networks

DEG in ENHCs against HCs or NECs were selected to search for interactions and molecular pathways using the Ingenuity Pathways Analysis software (IPA^®^)^2^ and STRING database^3^. Core analysis tool was executed using the DEG of each dataset. For both specific molecular pathways for ENHCs against HCs or NEC in the cochlea and the vestibular organs were generated and all with a *p*-value < 0.05 were considered as significant pathways for supporting cells. All pathways were manually inspected and molecular pathways not related with the inner ear tissue (i.e., cancer) were excluded for this study (Supplementary Tables S1–S4).

Networks of DEG were algorithmically generated based on their shortest connectivity and a score assigned by IPA. The top 25 networks were ranked with a score according to the number of nodes and edges involved in the network, but this may not be an indication of the quality or significance of the network. This score takes into account the number of focus genes in the network and the size of the network to approximate how relevant this network is to the original list of focus genes (Krämer et al., 2014). The genes found in the network that share diseases and functions terms in the four dataset were uploaded in STRING platform to identify new biological interactions (Szklarczyk et al., 2015).

## Results

We selected microarray and RNA-seq public datasets from the mouse cochlea and the utricle. All datasets are available in the gEAR website^4^ or in the original articles (Table 1).

The analysis of mouse gene expression data between ENHCs and HCs showed 1723 and 1559 DEG in the cochlear and vestibular tissues, respectively. However, only 865 genes were up-regulated in ENHCs in the organ of Corti and 660 genes were up-regulated in the vestibular maculae.

When we compared NECs vs. HCs, 2374 and 2283 DEG were found for cochlear and vestibular tissue respectively. Interestingly, 1129 genes were up-regulated in NECs in cochlea tissue and 1202 genes in the vestibular NECs. Finally we compared ENHCs and NECs to identify differences between both types of supporting cells. Our results showed 1120 and 1135 DEG for the cochlea and the vestibular organs, with 589 and 586 genes up-regulated in ENHCs in the cochlea and in the vestibular organs, respectively (Table 2).

**Table 2:** Differentially expressed genes found in the mouse inner ear.

	Cell type	Differentially expressed genes (N)	Up regulated genes (%)
Cochlea	ENHCs vs. HC	1723	865 (50.20)
	NEC vs. HC	2374	1129 (52.44)
	ENHCs vs. NEC	1120	589 (52.59)
Vestibular	ENHCs vs. HC	1559	660 (42.33)
	NEC vs. HC	2283	1210 (53.00)
	ENHCs vs. NEC	1135	586 (51.63)

*HC, Hair cells; ENHCs, Epithelial non-hair cells; NEC, Non-epithelial cells*.

### Pathways in Cochlear Supporting Cells

In the mouse cochlea, DEG obtained when ENHCs were compared to HCs showed a significant enrichment in eight molecular pathways. Particularly, two pathways were up-regulated, one was down-regulated and five were undetermined according to the z-score (Supplementary Table S1). The most significant pathway was “axonal guidance signaling” (AGS) with a *p* = 4.37 × 10^−8^, showing a 12% of the genes up-regulated and 3% down-regulated genes. DEG from ENHCs against NEC showed 14 pathways in which six pathways were up-regulated, two down-regulated and six undetermined (Supplementary Table S2). The most significant pathway was also “AGS” with a *p* = 1.04 × 10^−8^, showing a 6% of the genes up-regulated and 6% down-regulated genes.

The significant molecular pathways with >10% DEG linked with inner ear from cochlear supporting cells were ranked according to the percentage of DEG genes (Table 3). This table presents a significant enrichment in 18 pathways, highlighting “4-aminobutyrate (GABA) Degradation I” (*p* = 3.89 × 10^−4^) with a 66% of up-regulated and 33% of down-regulated genes, and “Calcium Transport I” (*p* = 3.80 × 10^−4^), showing a 50% of down-regulated genes when ENHCs were compared against HCs. In addition, “RhoGDI Signaling” was the top ranked pathway (*p* = 3.31 × 10^−8^), showing 8% of up-regulated and 8% of down-regulated genes when ENHCs were compared with NECs. Of note, we found the “AGS”, “Leukocyte Extravasation Signaling” and “Signaling by Rho Family GTPases” were pathways presented in both comparisons although with different number of DEG.

**Table 3:** Molecular pathways with >10% differentially expressed genes (DEG) in cochlear supporting cells.

		ENHCs vs. HCs		ENHCs vs. NECs	
Ingenuity canonical pathways	Genes (N)	Down	Up	Down	Up
4-aminobutyrate (GABA) degradation I	3	1 (33%)	2 (66%)	-	-
Calcium transport I	10	5 (50%)	0 (0%)	-	-
Inhibition of matrix metalloproteases	36	6 (2%)	8 (22%)	-	-
Calcium signaling	162	16 (10%)	8 (5%)	-	-
Axonal guidance signaling	433	14 (3%)	50 (12%)	27 (6%)	25 (6%)
Leukocyte extravasation signaling	198	3 (2%)	25 (13%)	12 (6%)	14 (7%)
Signaling by Rho family GTPases	242	6 (2%)	27 (11%)	15 (6%)	18 (7%)
RhoGDI signaling	168	-	-	14 (8%)	14 (8%)
Wnt/beta-catenin signaling	164	-	-	15 (9%)	9 (6%)
Regulation of the epithelial-mesenchymal transition pathway	182	-	-	11 (6%)	14 (8%)
Agranulocyte adhesion and diapedesis	156	-	-	11 (7%)	10 (6%)
Ephrin receptor signaling	170	-	-	10 (6%)	12 (7%)
Granulocyte adhesion and diapedesis	149	-	-	9 (6%)	11 (7%)
Thrombin signaling	198	-	-	11 (6%)	13 (7%)
RhoA signaling	116	-	-	7 (6%)	9 (8%)
Tight junction signaling	155	-	-	13 (8%)	6 (4%)
CREB signaling in neurons	176	-	-	6 (3%)	14 (8%)
Regulation of actin-based motility by Rho	82	-	-	5 (6%)	7 (9%)

*Pathways were identified using all DEG*.

### Pathways in Vestibular Supporting Cells

In the vestibular data, DEG from the ENHCs against HCs showed 13 pathways with an enrichment of genes (*p* ≤ 10^−4^). Particularly, five pathways were up-regulated, two down-regulated and six undetermined according to the z-score (Supplementary Table S3). The most significant pathway was “Leukocyte Extravasation Signaling” (*p* = 8.70 × 10^−6^) showing 13% of the genes up-regulated. DEG from ENHCs against NECs showed enrichment in 14 pathways with five of them up-regulated, one down-regulated and eight undetermined (Supplementary Table S4). From this comparison, we highlighted pathways such as “AGS” (*p* = 5.01 × 10^−8^) and “Leukocyte Extravasation Signaling” (*p* = 8.91 × 10^−7^) that presented 7% of up-regulated genes and 8% down-regulated. The significant pathways with >10% DEG in the vestibular datasets and linked with inner ear from both tables were combined and ranked according to the percentage of DEG genes (Table 4). This table presents a significant enrichment in 20 pathways. In ENHCs against HCs highlighted “Calcium Transport I” (*p* = 2 × 10^−4^) with a 10% of up-regulated and 40% of down-regulated genes, and “Glutathione Redox Reactions I” (*p* = 10^−4^), showing a 26% up-regulated genes. When ENHCs were compared against NECs, “RhoGDI Signaling” was the most significant pathway (*p* = 9 × 10^−4^), showing 7% of up-regulated and 5% of down-regulated genes. In addition we found the “AGS”, “Leukocyte Extravasation Signaling” and “Signaling by Rho Family GTPases” were pathways presented in both comparisons although with a different number of DEG.

**Table 4:** Significant pathways with >10% differentially expressed genes (DEG) in vestibular supporting cells.

		ENHCs vs. HCs		ENHCs vs. NECs	
Ingenuity canonical pathways	Genes (N)	Down	Up	Down	Up
Calcium transport I	10	4 (40%)	1 (10%)	-	-
Glutathione redox reactions I	19	2 (11%)	5 (26%)	-	-
Inhibition of matrix metalloproteases	36	1 (3%)	8 (22%)	-	-
Calcium signaling	162	16 (10%)	10 (6%)	-	-
Rac signaling	115	8 (7%)	10 (9%)	-	-
RhoA signaling	116	6 (5%)	12 (10%)	-	-
Leukocyte extravasation signaling	198	4 (2%)	26 (13%)	15 (8%)	13 (7%)
Signaling by Rho family GTPases	242	10 (4%)	24 (10%)	10 (4%)	15 (6%)
Axonal guidance signaling	433	13 (3%)	35 (8%)	22 (5%)	28 (6%)
Clathrin-mediated endocytosis signaling	183	9 (5%)	17 (9%)	-	-
ILK signaling	181	3 (2%)	21 (12%)	-	-
Glutathione-mediated detoxification	24	1 (4%)	6 (25%)	-	-
eNOS signaling	164	8 (5%)	14 (9%)	-	-
RhoGDI signaling	168	-	-	8 (5%)	11 (7%)
Wnt/beta-catenin signaling	164	-	-	11 (7%)	13 (8%)
Regulation of the epithelial-mesenchymal transition pathway	182	-	-	8 (4%)	17 (9%)
Granulocyte adhesion and diapedesis	149	-	-	9 (6%)	12 (8%)
Tight junction signaling	155	-	-	15 (10%)	5 (3%)
Gap junction signaling	185	-	-	8 (4%)	13 (7%)
G alpha i signaling	116	-		5 (4%)	10 (9%)

*Pathways were identified using all DEG*.

### Shared Pathways in the Cochlear and Vestibular Datasets

**Figure 3:**
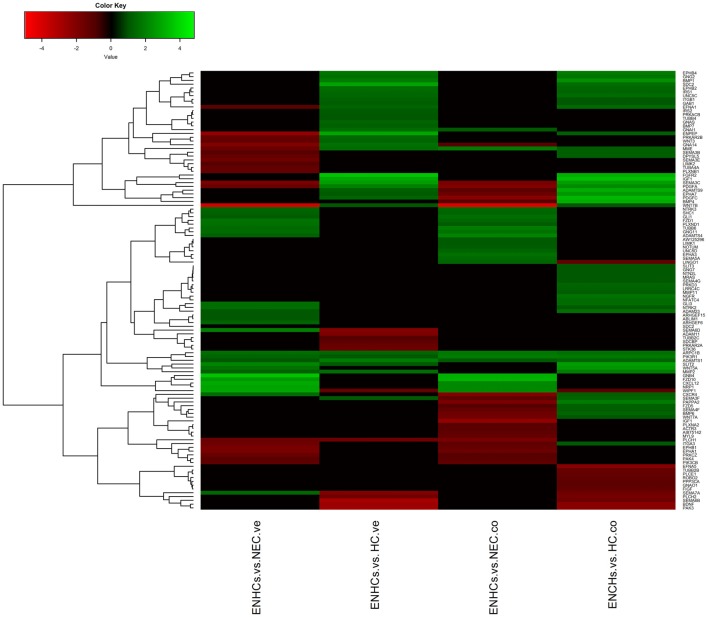
Heatmaps showing gene expression profiles in ENCHs compared with NECs and HCs in the vestibular and cochlear datasets. Differentially expressed genes (DEGs) in “Axonal Guidance Signaling” pathway.

**Figure 4:**
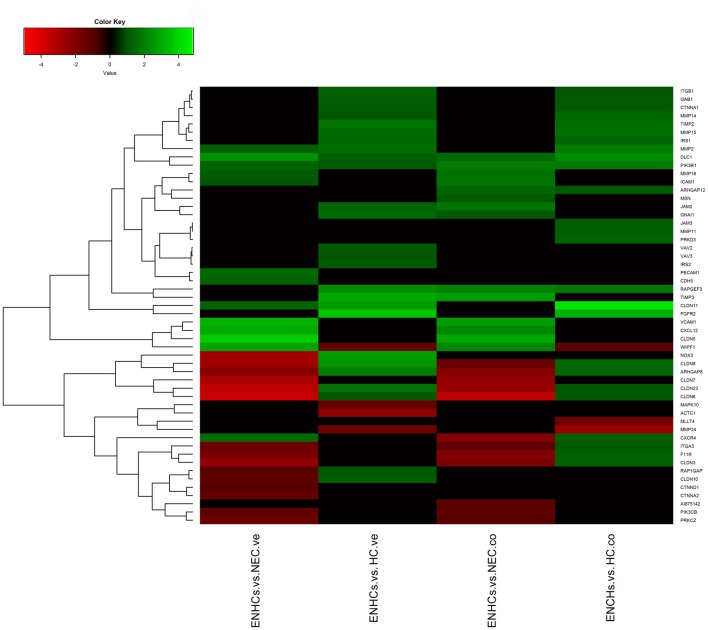
Heatmaps showing gene expression profiles in ENCHs compared with NECs and HCs in the vestibular and cochlear datasets. DEGs in “Leukocyte Extravasation Signaling” pathway.

**Figure 5:**
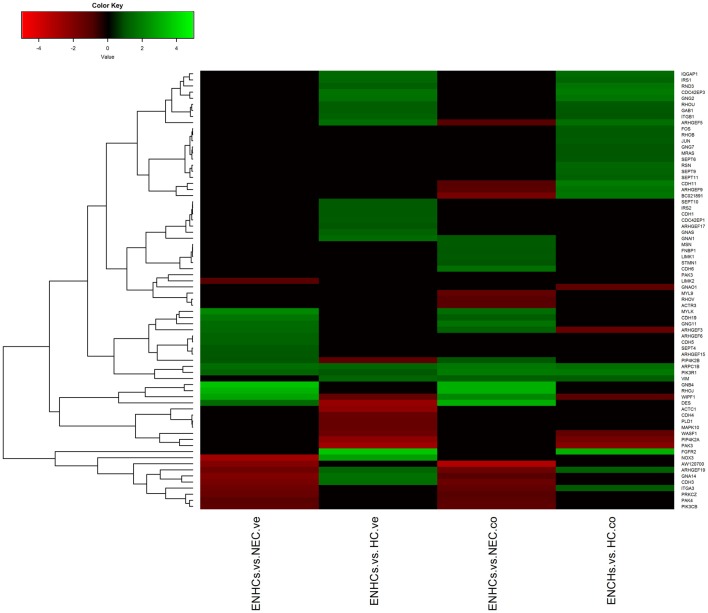
Heatmaps showing gene expression profiles in ENCHs compared with NECs and HCs in the vestibular and cochlear datasets. DEGs in “Signaling by Rho Family GTPases” pathway.

In both tissues we found three common pathways: (1) “Leukocyte Extravasation Signaling”; (2) “Signaling by Rho Family GTPases”; and (3) “AGS”. However, the genes revealed specific expression patterns, according to the cellular subtype and location (Figures 3–5). So, “AGS” was the top ranked pathway in cochlear ENHCs when they were compared to cochlear HCs (Supplementary Figure S1); “Leukocyte Extravasation Signaling” was the top ranked pathway in vestibular ENHCs compared to HCs (Supplementary Figure S2) and “Signaling by Rho Family GTPases” was the most relevant pathway when ENHCs were compared to HC in the cochlea (Supplementary Figures S3).

In addition, DEG datasets in supporting cells were uploaded in STRING platform to search for additional molecular pathways, according to KEGG database. Remarkably, all the datasets showed enrichment in three pathways: (1) “Focal Adhesion” (Supplementary Figure S4); (2) “PI3K-Akt signaling” (Supplementary Figure S5); and (3) “extracellular matrix (ECM)-receptor interaction” (Supplementary Figure S6), although these pathways also have more DEG genes in the vestibular than in the cochlear datasets. Moreover the “AGS” only was found to be enriched in cochlear ENHCs (*p* = 6.37 × 10^−5^; Supplementary Tables S5, S6).

### Shared Networks for Cochlear and Vestibular Datasets

By using IPA, we generated and ranked 25 networks for each dataset with diverse diseases and functions, the most biologically significant being the network associated with “Auditory and Vestibular System Development and Function” term that were found in all the dataset comparisons (Supplementary Table S7). The 126 DEG genes detected in any of the fourth dataset with “auditory and vestibular system development and function” term were uploaded in STRING to visualize their biological interactions (Supplementary Figure S7).

### Specific Genes in Cochlear and Vestibular Supporting Cells

**Figure 6:**
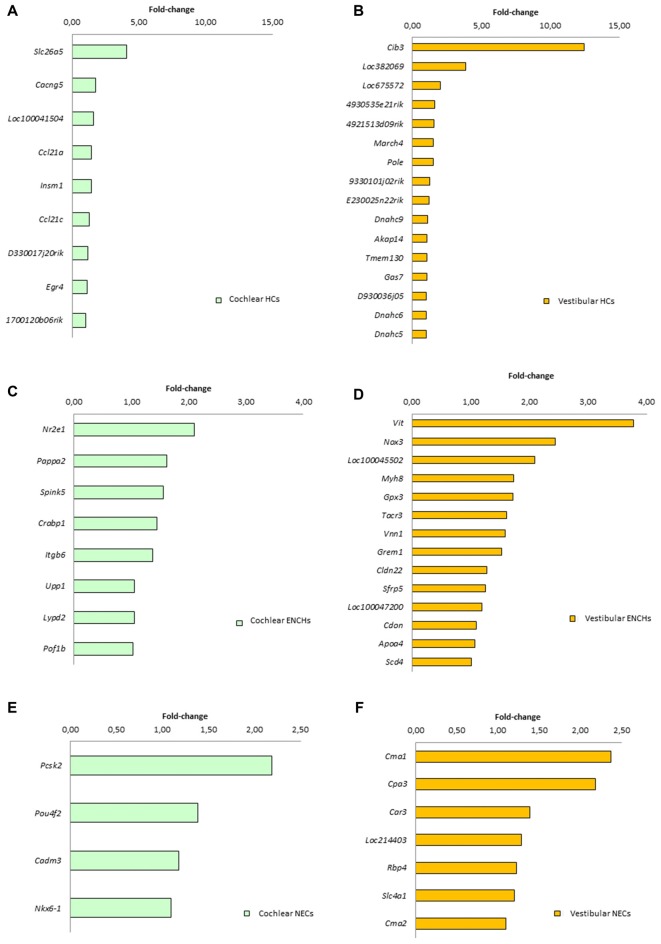
Specific marker genes for each subtype found in the cochlea (left panel, green) and vestibular organs (right panel, orange). **(A)** Cochlear HCs. **(B)** Vestibular HCs. **(C)** Cochlear ENCHs. **(D)** Vestibular ENCHs. **(E)** Cochlear NECs. **(F)** Vestibular NECs.

We searched for specific genes only expressed in each cell subtype and tissue. There were 9 genes for HCs, 8 for ENHCs and 4 for NEC in the cochlea, whereas there were 16 genes for HCs 14 for ENHCs and seven for NEC in the vestibular datasets (Figure 6). In addition, we identified common marker genes for each cell subtype in the organ of Corti and vestibular maculae. We found 107 specific genes in HCs including the *ATOH1* as the most representative, eight in ENHCs and 62 in NECs (Supplementary Table S8).

In addition, each cell type from the vestibular dataset was compared against cochlear datasets. Raw data and significant pathways are presented in Supplementary Tables S9–S11 (S9, HCs; S10, ENHC; S11, NEC). Remarkably, vestibular ENHCs showed an enrichment in 22 pathways when they were compared with cochlear ENHCs (*p* < 0.001); however, only two pathways were found to be significantly different between vestibular and cochlear HCs.

### Gene Expression Shows Tonotopy in Supporting Cells

**Figure 7:**
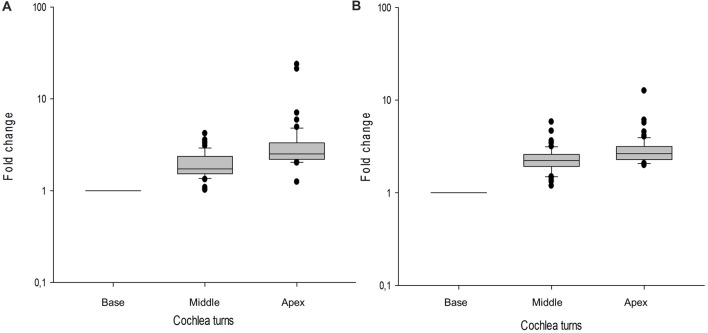
Box-plots showing gene expression profile across the basal, middle and apical turns of the mouse cochlea shows tonotopy. Values of each gene are indicated as a relative value to the basal turn. **(A)** ENCHs against HCs, **(B)** ENCHs against NECs. Error bar represent standard deviation.

In the cochlear dataset, 66 genes showed a tonotopic expression in at least one of the cochlear turns when ENHCs were compared with HCs. Moreover, 81 genes were differentially expressed between ENHCs and NECs, according to the cochlear turn (Supplementary Table S12). From these data, 37 DEG in ENHCs compared with HCs showed a tonotopic expression gradient and genes such as *Tectb* and *Fst* showed the highest fold-change between apex and base (23.85 and 21.20, respectively; Figure 7A). Despite the fact that 61 genes also showed a tonotopic expression pattern when ENHCs were compared to NECs, they exhibited less difference in the expression gradient than ENHCs compared to HCs (Figure 7B).

## Discussion

These results pointed that six main molecular pathways were found in ENHCs in the cochlea and vestibular datasets that could define the essential processes for supporting cells: (1) “Leukocyte Extravasation Signaling”; (2) “Signaling by Rho Family GTPases”; (3) “AGS”; (4) “Focal Adhesion”; (5) “PI3K-Akt signaling”; and (6) “ECM-receptor interaction”. It is well known that ENHCs further contribute to inner ear homeostasis by forming an active epithelial barrier with HCs to regulate ions fluxes between endolymph and perilymph (Bird et al., 2010) and the pathways found related to intercellular connections such as “Focal Adhesion” or “PI3K-Akt signaling” and cell-ECM adhesion as “ECM-receptor interaction” confirms it.

In addition, the role of ENHCs in the elimination damaged HCs thought phagocytosis and Rho kinases have been demonstrated for previous studies and predicted by our analyses that show that “Signaling by Rho Family GTPases” is involved. More recent studies have shown that macrophages are recruited after HCs injury, however the mechanism to attract macrophages to sites of HCs injury remains unknown (Kaur et al., 2015). Our results reveal that ENHCs may play this role overexpressing genes associated with transmigration as *Calm1* and *Jam2* inner ear maturation. In this stage, only 13% and 7% pathway genes are active, but the number of genes active could increase after the HC injury.

However, the most relevant finding of our results is that ENHCs have an unexpected role as axonal guidance towards nerve fibers during the maturation of the mouse inner ear. So this results show that the paracrine signaling that regulates the innervation of the sensory epithelium is not only limited to HCs (Bianchi et al., 2005; Yang et al., 2011), and supporting cells also participate in this process. So, the comparison of ENHCs against HCs in the cochlea found that the pathway “AGS” had a 15% of the genes up or down-regulated. This supports the hypothesis that both cochlear and vestibular supporting cells are contributing to the axonal guiding and the maintenance of afferent and efferent innervation of the sensorineural epithelia (Appler and Goodrich, 2011).

In addition, “4-aminobutyrate (GABA) Degradation I”, “Calcium Transport I” and “Calcium Signaling” showed a significant enrichment of DEG. However, each pathway showed a different function. So, DEGs in the “4-aminobutyrate (GABA) Degradation I pathway” showed that ENHCs have similar level the GABA transporter that HCs, but the degradation enzymes were highly expressed, indicating that this pathway should play a role in the regulation of neurotransmitter turnover in supporting cells. This idea is supported because both cochlear and vestibular supporting cells express the Glutamate Aspartate Transporter (GLAST) at their basolateral membranes, and supporting cells regulate the uptake of the excitatory neurotransmitter glutamate (Furness and Lehre, 1997; Takumi et al., 1997; Wan et al., 2013). Both results suggest that ENHCs would control the synaptic gap levels of glutamate and GABA, as it has been described in astrocytes (Robinson and Jackson, 2016). This mechanism could protect afferent dendrites of the neurotransmitters excess produced during noise overstimulation (Robertson, 1983; Liberman, 2017), exposure to aminoglycoside antibiotics (Mao-Li, 2009) or ischemeia (Puel et al., 1994; Tabuchi et al., 2010).

On the other hand, the pathways “Regulation of the Epithelial-mesenchymal Transition Pathway” along with “Signaling by Rho Family GTPases” showed and enrichment of DEGs in the cochlear datasets. This two pathways revealed that ENHCs in the neonatal mouse still have the capacity to transdifferentiate into another cell type, unlike what occurs in the human inner ear (Maass et al., 2013).

When ENHCs were compared against HC in the vestibular dataset, one of the most representative enriched pathways was “ILK Signaling”, which acts as important regulator of integrins, transmembrane receptors that bind to the ECM and provide the structural framework for the formation of tissues. The ECM binds to substrate adhesion molecules on the surface of cells and influences various intracellular signaling pathways that regulate survival, proliferation, polarity and differentiation. In this pathway, MYH14 in one of the up regulated genes that is linked with sensorineural hearing loss without vestibular involvement (Donaudy et al., 2004). In addition, ILK activity is crucial for maintaining upstream signaling to Beta1-integrins and downstream signaling to AKT, GSK3 and PHI1 at the focal adhesion plaques (Velling et al., 2004). These events promote survival by inhibiting BAD, caspase 3/9 and cell cycle transition by blocking proteolysis of Cyclin D1. Other downstream targets of ILK-induced AKT include mTOR and NF-KB. ILK phosphorylation of GSK3-beta also inhibits Beta-catenin and Lymphoid enhancer factor/T cell factor proteins (LEF/TCF) interaction, which in turn regulates the function of Myc, cadherins and cAMP-response-element-binding protein (CREB). ILK may directly activate CREB through the ERK-MSK-1/2 signaling route (Wu and Dedhar, 2001). Interestingly “CREB Signaling in Neurons” was a significant pathway predicted in the cochlear ENCHs vs. NECs comparison.

We have also described the common marker genes between each cell subtype from the cochlea and the vestibular datasets. Our result found 107 marker genes in HCs, eight markers in ENHCs and 62 in NECs that indicates that cochlear and vestibular supporting cells clearly differ between each other; however, HCs and NECs have more common DEGs. So HCs and NECs would share some functions whereas ENHCs could play a different role, according if they are located in the organ of Corti or in the vestibular organ.

Tonotopy always have been associated with HCs (Ricci et al., 2003; Fettiplace, 2017), but our results show that also supporting cells show a gene expression pattern associated with tonotopy. In total, 37 DEG in ENHCs compared with HCs showed a tonotopic expression gradient. *Tectb* and *Fst* showed the highest fold-change between apical and basal turns. Previous studies have reported the role of these genes in tonotopy; *Tectb* is associated with low frequency hearing loss (Russell et al., 2007) and the complex *Activin/Fst* is involved in tonotopic organization of the mouse cochlea (Son et al., 2015). Since gene expression datasets were obtained from P1 and adult mice, only five genes showed tonotopy in both studies (Yoshimura et al., 2014; Waldhaus et al., 2015); *Fst from ENCHs* again HCs and *Pdgfra* (Liu and Davis, 2014), *Pvalb* (Moore and Wehr, 2013), *Dner* (Kowalik and Hudspeth, 2011), *Calb2* (Liu and Davis, 2014) from ENCHs again HCs.

The supporting cell genes found when ENHCs were compared against HCs or NECs were *Vim*, *Sdpr* (also named *Cavin-2)* and *Spock3* showed a tonotopic expression pattern. Previous studies have demonstrated that *Vim* (Arnold and Anniko, 1990), and *Spock3* (Boopathy et al., 2017) are linked with the tonotopy whereas *Sdpr* is associated with caveole formation and therefore with detergent-resistant membrane regions that host 11 molecules as Connexin 43 *Gjb6*, *Kcnq1*, or *Tecta* (Thomas et al., 2014). The gene *Sdpr* is linked with eNOS pathway (Boopathy et al., 2017) and it has been previously related with pathological changes in the inner ear (Zhu et al., 2008). In addition, *Serpinf1* and *Otor* that had more expression in the base than in the apex have a role in the functional maturation of the mechanoelectrical transduction machinery (Scheffer et al., 2015).

Waldhaus et al. (2015) obtained gene expression data from Deiters cell rows 1 and 2 (DC1/2), GER, IPC, IPHs and OPC. Interestingly, 12 DEGs were shared among these different cell types and the cochlear ENHCs against HCs dataset obtained by Elkon et al., 2015 (*Otor, Gli3, Dkk3, Sox9, Grb10, Ccnd1, Fos, Dnmt3a, Wnt7a, Epha7, Efna1, Wnt7b)*. These 12 genes illustrate the role of ENHCs in the maturation and elongation of cochlea.

In the comparison of ENHCs against NECs only three genes (*Otor, Gdf10, Tgfbr2*) were exclusively detected in the cochlear supporting cells. *Otor* gene was also found in DC1/2, and *Gdf10* and *Tgfbr2* in OPC. All these genes showed more expression in the base than in the apex linked with their role in the functional maturation of the organ of Corti (Ficker et al., 2004; Liu et al., 2015; Scheffer et al., 2015).

The limitation of this study is that it is entirely based on bioinformatics analyses. So, an experimental validation of these datasets using single cell RNAseq or FISH on cochlear and vestibular ENHCs would be necessary to confirm our findings. So, we have predicted 64 dysregulated genes in AGS pathway in the cochlea (Supplementary Table S1) and Waldhaus et al. (2015) already validated 12 of these 64 genes by qPCR. The validation of the three top ranked pathways for the comparison in Supplementary Tables S1–S4 will involve several 100 genes and a collaborative study with other laboratories.

## Conclusion

We have predicted the main pathways and molecular networks with several protein clusters for non-sensory epithelial cells of the mouse organ of Corti and vestibular neuroepithelium of the utricle. These pathways will facilitate the design of a molecular map in supporting cells to conduct functional studies of novel candidate genes for hearing or vestibular loss associated with in non-sensory epithelial cells such as Meniere Disease.

## Author Contributions

TR and JAL-E designed the study and drafted the manuscript. TR and AG-M performed data extraction, bioinformatics analyses and molecular networks generation. TR, AG-M and JAL-E revised and approved the final version of the manuscript.

## Conflict of Interest Statement

The authors declare that the research was conducted in the absence of any commercial or financial relationships that could be construed as a potential conflict of interest.

## Footnotes

^1^http://umgear.org/index.html

^2^http://www.ingenuity.com/products/ipa

^3^https://string-db.org/

^4^http://umgear.org/index.html
