# Supplementary material for: Bioinformatic Integration of Molecular Networks and Major Pathways Involved in Mice Cochlear and Vestibular Supporting Cells
Source: Front Mol Neurosci. 2018 Apr 5;11:108. doi: 10.3389/fnmol.2018.00108 (PMC5895758; doi:10.3389/fnmol.2018.00108)
Supplement: Supplementary file 2 [file Data_Sheet_1.pdf]

Supplementary Material

Bioinformatic Integration of Molecular Networks and Major Pathways Involved in Mice Cochlear and Vestibular Supporting Cells

Teresa Requena[1*], Alvaro Gallego-Martinez[1] and Jose A. Lopez-Escamez[1,2,3]

*** Correspondence:** Corresponding Authors: mariateresa.requena@genyo.es

Supplementary Figures legends

Supplementary Figure 1 Axonal Guidance Signaling pathway in mouse cochlear ENHCs (supporting cells), according to IPA. Up and down-regulated genes are shown in red and green, respectively. Each type of molecule is represented according to a functional coding detailed below.

Supplementary Figure 2 Leukocyte Extravasation Signaling pathway in mouse vestibular ENHCs, according to IPA. Up and down-regulated genes are shown in red and green, respectively. Each type of molecule is represented according to a functional coding detailed below.

Supplementary Figure 3 Rho Family GTPases pathway in mouse cochlear ENHCs, according to IPA. Up and down-regulated genes are shown in red and green, respectively. Each type of molecule is represented according to a functional coding detailed below.

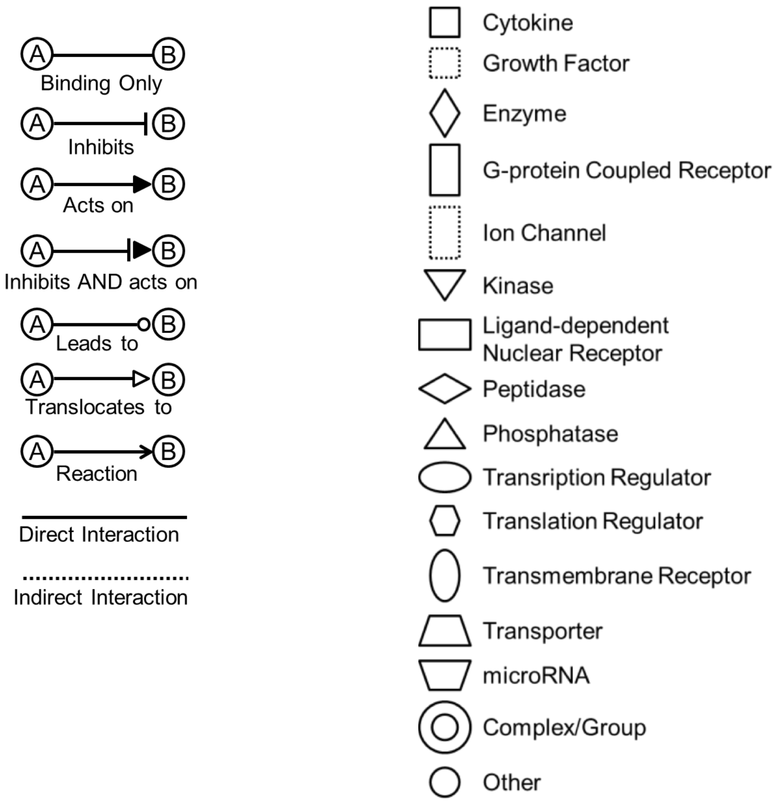

Supplementary Figure 4 Focal Adhesion pathway in mouse cochlear and vestibular supporting cells, according to KEGG.

Supplementary Figure 5 PI3K-Akt signaling pathways in mouse cochlear and vestibular supporting cells according to KEGG.

Supplementary Figure 6 Extracellular matrix (ECM)-receptor interaction according to KEGG.

Supplementary Figure 7 Network with biological interactions generated by STRING using all the genes with 'auditory and vestibular system development and function" from IPA.
