# Supplementary figures and images for: Bioinformatic Integration of Molecular Networks and Major Pathways Involved in Mice Cochlear and Vestibular Supporting Cells
Source: Front Mol Neurosci. 2018 Apr 5;11:108. doi: 10.3389/fnmol.2018.00108 (PMC5895758; doi:10.3389/fnmol.2018.00108)

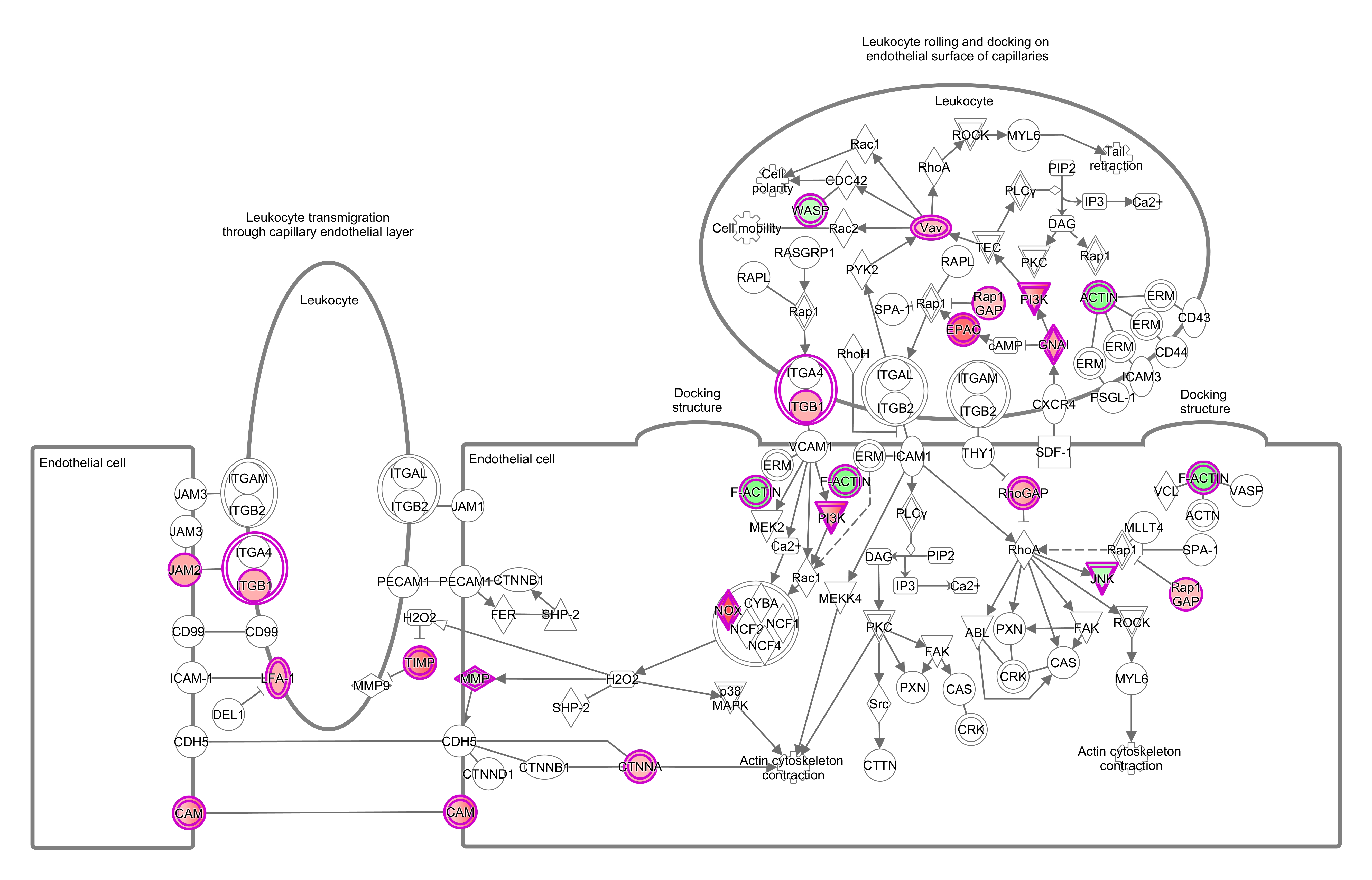

Supplement: Supplementary file 4 [file Image_2.TIF]

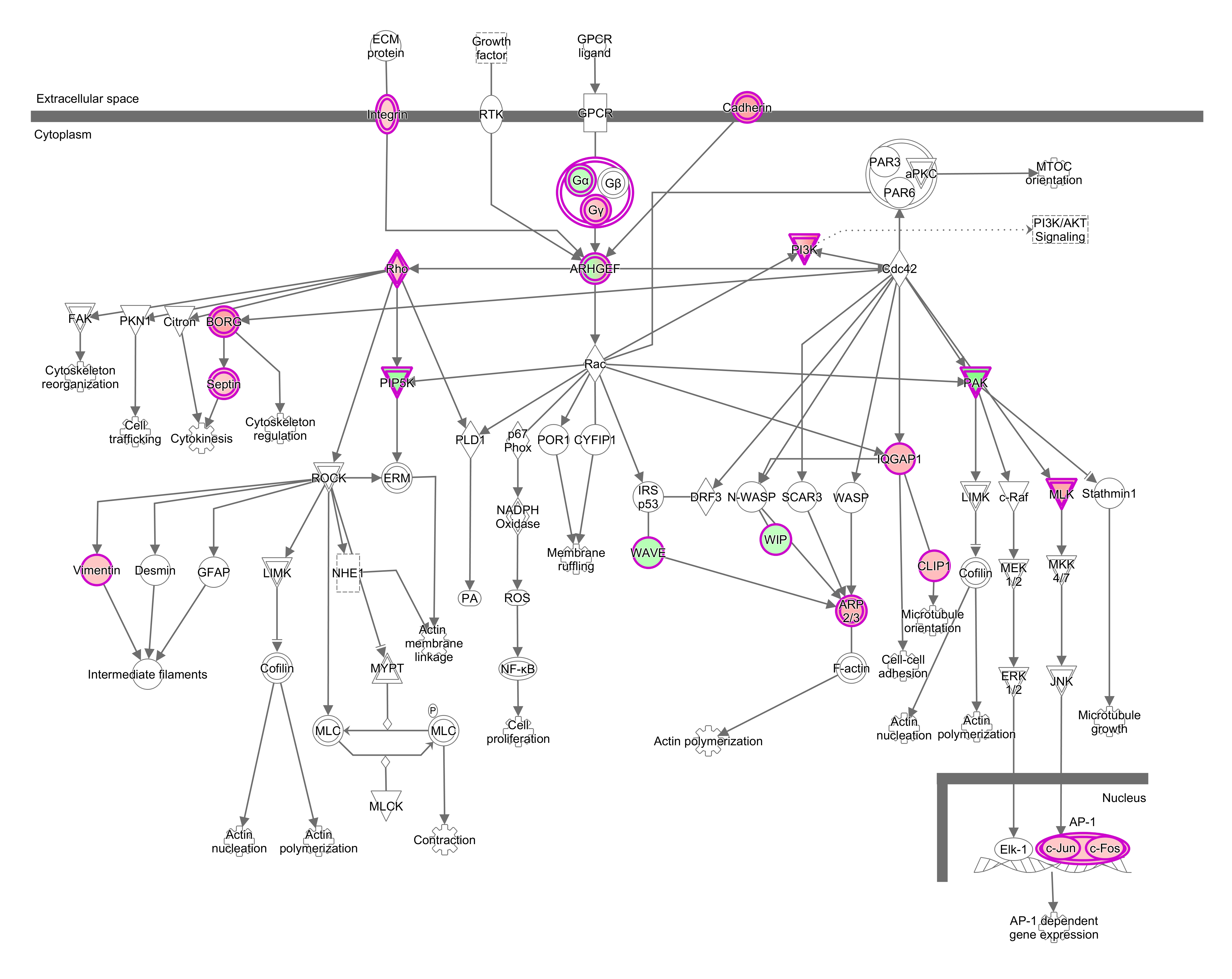

Supplement: Supplementary file 5 [file Image_3.TIF]

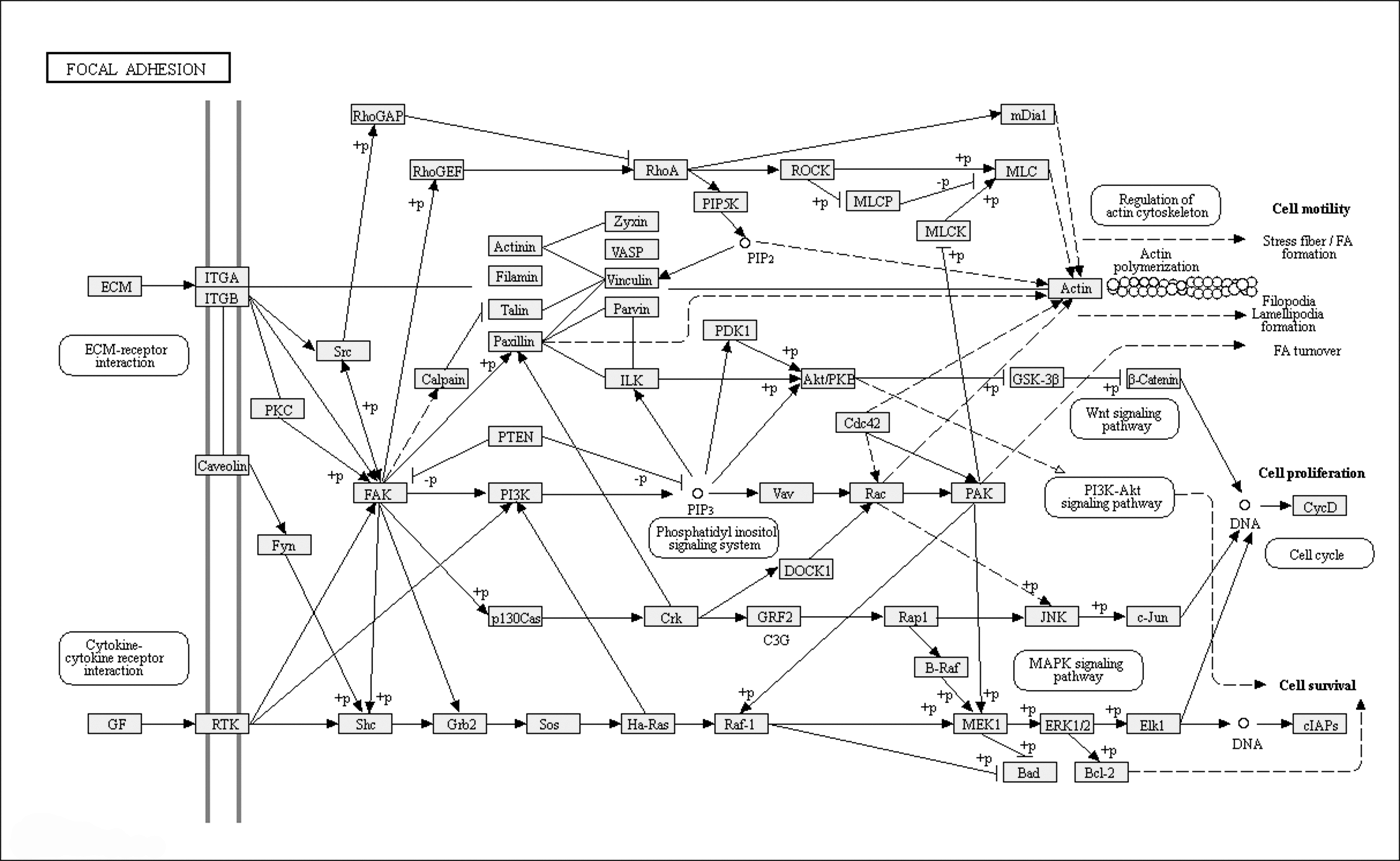

Supplement: Supplementary file 6 [file Image_4.TIF]
